# Alternative splicing of TGF-betas and their high-affinity receptors TβRI, TβRII and TβRIII (betaglycan) reveal new variants in human prostatic cells

**DOI:** 10.1186/1471-2164-8-318

**Published:** 2007-09-11

**Authors:** Lutz Konrad, Jonas A Scheiber, Elke Völck-Badouin, Marcel M Keilani, Leslie Laible, Heidrun Brandt, Ansgar Schmidt, Gerhard Aumüller, Rainer Hofmann

**Affiliations:** 1 Department of Urology, Medical Faculty, 35033 Marburg, Germany; 2 Department of Anatomy and Cell Biology, Medical Faculty, 35033 Marburg, Germany; 3 Department of Pathology, Medical Faculty, 35033 Marburg, Germany

## Abstract

**Background:**

The transforming growth factors (TGF)-β, TGF-β1, TGF-β2 and TGF-β3, and their receptors [TβRI, TβRII, TβRIII (betaglycan)] elicit pleiotropic functions in the prostate. Although expression of the ligands and receptors have been investigated, the splice variants have never been analyzed. We therefore have analyzed all ligands, the receptors and the splice variants TβRIB, TβRIIB and TGF-β2B in human prostatic cells.

**Results:**

Interestingly, a novel human receptor transcript TβRIIC was identified, encoding additional 36 amino acids in the extracellular domain, that is expressed in the prostatic cancer cells PC-3, stromal hPCPs, and other human tissues. Furthermore, the receptor variant TβRIB with four additional amino acids was identified also in human. Expression of the variant TβRIIB was found in all prostate cell lines studied with a preferential localization in epithelial cells in some human prostatic glands. Similarly, we observed localization of TβRIIC and TGF-β2B mainly in the epithelial cells with a preferential localization of TGF-β2B in the apical cell compartment. Whereas in the androgen-independent hPCPs and PC-3 cells all TGF-β ligands and receptors are expressed, the androgen-dependent LNCaP cells failed to express all ligands. Additionally, stimulation of PC-3 cells with TGF-β2 resulted in a significant and strong increase in secretion of plasminogen activator inhibitor-1 (PAI-1) with a major participation of TβRII.

**Conclusion:**

In general, expression of the splice variants was more heterogeneous in contrast to the well-known isoforms. The identification of the splice variants TβRIB and the novel isoform TβRIIC in man clearly contributes to the growing complexity of the TGF-β family.

## Background

Transforming growth factor (TGF)-β is a secreted cytokine implicated in a wide variety of biological processes such as apoptosis, motility, tumorigenesis, proliferation, differentiation, and gene expression [1]. In mammals three TGF-betas, TGF-β1, TGF-β2 and TGF-β3, have been cloned, and although they show very often overlapping functions in vitro, the isoform-specific knockout mice revealed non-redundant and non-overlapping phenotypes. Approximately 50% of the TGF-β1 knockout mice died during embryogenesis from yolk sac defects; the survivors developed inflammatory disorders and died typically within one month [2-4]. TGF-β2 knockout mice were perinatally lethal and developed defects in different organs such as heart, kidney, testis, as well as various craniofacial defects and axial and appendicular skeletal defects [5]. Moreover, TGF-β3 knockout mice were perinatally lethal due to a delayed lung development and defective palatogenesis [6,7].

Secretion and inactivation of the TGF-betas is regulated by the latency-associated peptides (LAPs) which are generated from the respective N-terminal TGF-β proteins by cleavage [8]. Additionally, latent TGF-β binding proteins (LTBP1-4) are covalently attached to the LAPs and mediate storage in the extracellular matrix (ECM). After activation by proteolytic enzymes or acidic environment the TGF-betas bind with high affinity to the serine/threonine kinase receptor TβRII which in turns phosphorylates TβRI. Activation of the receptor complex propagates the signal downstream to the Smad proteins, who regulate many TGF-β-induced transcriptional responses [9]. Alternatively, TGF-β2 can bind to the accessory receptor TβRIII, who facilitates binding of TGF-β2 to TβRII. However, signal transduction is initiated again by TβRI. Recently, it was shown that TGF-β2 might also bind to an alternative splice product of the TβRII gene, mainly expressed in osteoblasts and mesenchymal cells. The receptor isoform TβRIIB binds TGF-β2 also in the absence of TβRIII and then activates TβRI starting the signal transduction [10]. However, recently it was shown in human chondrocytes that TβRIIB exerted a higher affinity for TGF-β1 than for TGF-β2 [11]. In addition to alternative splicing of TβRII, TGF-β2 and TβRI also were demonstrated to be alternatively spliced in human prostatic PC-3 cells [12] and embryonic stem cells from mouse [13], respectively.

TGF-betas are believed to be involved in several aspects of carcinogenesis. At the beginning of tumor formation the TGF-betas might inhibit proliferation of cancer cells, but with ongoing dedifferentiation, the TGF-betas and the receptors seem to enhance motility and metastasis of the tumor cells [1,14]. In more advanced and especially metastasised stages higher serum levels of TGF-β1 were found [15] and reduced expression of TβRII and TβRI in the tumor tissue was associated with poor prognosis [16].

Recently, analyses of alternative splicing indicated that approximately 40–60% of human genes express splice variants, suggesting that alternative splicing contributes to the growing complexity of the human genome [17]. In many transcripts, addition or deletion of complete exons or introduction of an early stop codon may result in a truncated or unstable mRNA [18]. Alternative splicing has been shown to be involved in ligand binding to growth factor receptors like TβRIIB [10], cell adhesion or various human diseases [19]. Additionally, alternative splicing occurs sometimes during developmental processes and may be restricted to distinct tissues [18]. Interestingly, it was reported that more alternative splicing was found in organs such as testis, pancreas, placenta, and liver [20]. Up to date many groups have presented genomic analyses of alternative splicing by use of expressed sequence tags (EST, [e.g. [21-23]] or microarrays [20]. Most of these results are now available in databases [17].

In this study, we have analyzed the mRNA expression of the TGF-betas and the receptors TβRI-III mainly in human prostatic cells available to us and identified the splice variants TGF-β2B, TβRIB, TβRIIB and the new variant TβRIIC. Of note, the alternatively spliced exons were found in the N-terminal part of the proteins and extracellular domains of the receptors. The splice variant TGF-β2B could be identified in more species than the other isoforms and showed less sequence variation among the various species. Furthermore, this is the first report showing localization of the splice variants TβRIIB, TβRIIC and TGF-β2B in human prostate tissue.

## Results

### Literature and database search for alternative splicing

The search in the literature (PubMed) and sequence databases for TGF-betas and their high-affinity receptors displayed deleted or additional exons. Alternative splicing of the TGF-beta ligands was described for TGF-β1 in pig [24] and for TGF-β2 in human and rat [12,25]. Alternative splicing of the high-affinity receptors was demonstrated for TβRI in mouse, rat and boar [13,26,27], and for TβRII in mouse and human [28-30].

In the database ASDB [21], dealing with alternative splicing, TGF-β2 and TGF-β3 were mentioned to contain splice variants, and in the database ASAP [31] three isoforms for TGF-β1 were described. The database EASED [32] showed many but not all of the aberrant ESTs which were found in this study.

### Alternative splicing and mRNA expression of TβRI

**Figure 1:**
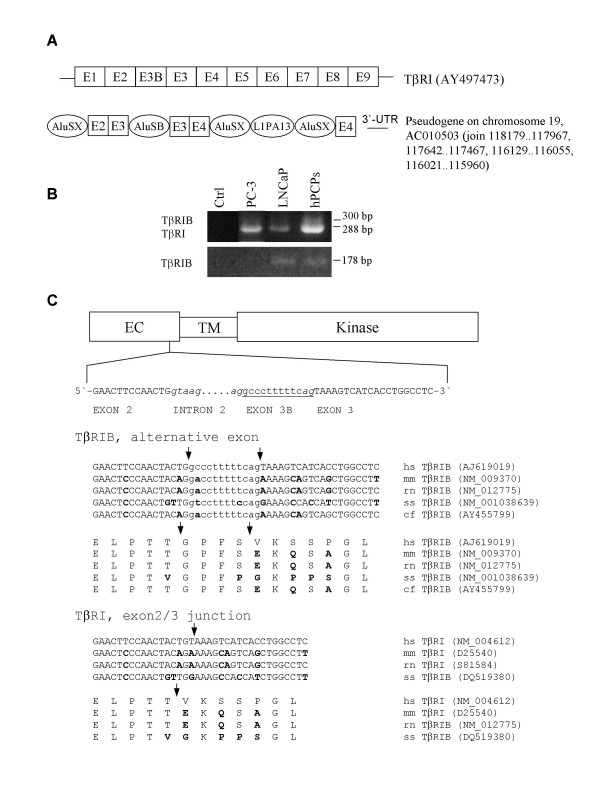
(**A**) Comparison of the exon structure of the human TβRI mRNA with the pseudogene on chromosome 19. A detailed alignment of the pseudogene with exons 2 to 4 and the 3'-UTR is available from the authors upon request. Lines depict the 5'-UTR and 3'-UTR. The repetitive elements AluSX, AluSB and L1Pa13 are encircled. (**B**) Expression pattern of the TβRI gene in human prostatic cells. Expression of both transcript variants (upper panel, 5-TB1RL/3-TB1RL) and expression of the splice variant TβRIB (lower panel; 5-TB1RL/3-HTB1RL) is demonstrated. (**C**) Scheme of the TβRI protein (EC, extracellular domain; TM, transmembrane domain; kinase, Ser/Thr kinase domain) with the nucleotide and amino acid sequence of exon 2 and exon 3 (capital letters) and the alternatively spliced exon 3B (lower case letters). Additionally, the partial sequence without the alternatively spliced exon is given below. The sequence of TβRI was not available for canis familiaris. The splice site junctions are indicated by italic letters. Bold letters mark the amino acid and nucleotide exchanges with respect to the human sequence. The accession numbers are given below (hs, homo sapiens; mm, mus musculus; rn, rattus norvegicus; ss, sus scrofa; cf, canis familiaris). Arrows indicate the exon boundaries. Ctrl, control.

Alignment of the human ESTs with the genomic sequence of the TβRI revealed several irregular ESTs but none of them with additional exons. We also identified a pseudogene of the TβRI gene on chromosome 19 reaching from exon 2 to exon 4 and a short stretch of 62 base pairs (bp) from the 3'-UTR (Fig. 1A). The pseudogene showed 87 sequence aberrations in 550 bp (16%) compared to the TβRI cDNA.

A 12 bp extension of the third exon of the TβRI was first found in mouse and rat [13,26] and recently in boars [27], but not in human. Therefore, we cloned both variants of TβRI, with and without the 12 bp extension (Acc. Nos. AJ619019 and AJ619020, respectively) from the human stromal cells hPCPs [33] (Figs. 1A,B). mRNA expression of TβRI and TβRIB was found in the stromal hPCPs cells and epithelial LNCaP cells, whereas PC-3 cells only expressed TβRI and not TβRIB (Fig. 1B). Because the splice variant TβRIB was only weakly expressed, we obtained improved PCR results by using a primer containing the 12 bp extension (Fig. 1B, Table 1). The additional exon 3B codes for 4 amino acids in the extracellular domain of the receptor and is also found in dogs and pigs (Fig. 1C). Up to date, the sequence of TβRI is not available for Canis familiaris (Fig. 1C). According to the exon classification [34] exon 3B with the 12 bp extension belongs to the exon-type with internal acceptor sites.

**Table 1:** Primer pairs used for characterization

**Gene (Acc No)^a^**	**Position**	**Designation**	**Size**	**Sequence**	**AT^b^**
TβRI, human,	256–275^c^	5-TB1RL	288 bp	GACCACAGACAAAGTTATAC	60°C
(NM_004612)	524–543	3-TB1RL	300 bp	TGGTGAATGACAGTGCGGTT	
(AJ619019)	159–178	3-HTB1RL	178 bp	TACTGAAAAAGGGCCAGTAG	52°C
					
TβRII, human	435–454	5-HTBR2B	274 bp	CGCGTATCGCCAGCACGATC	63°C
(NM_003242)	688–708	3-HTBR2B	349 bp	TGGTAGGGGAGCTTGGGGTCA	
(NM_001024847)	795–815	5-HTBR2E3	298 bp	GTAGCTCTGATGAGTGCAATG	60°C
	1072–1092	3-HTBR2E4	406 bp	TGGTTGATGTTGTTGGCACAC	
TβRIIC, human	89–108	5-HTBR2Z	319 bp	GGAGCACTTGTCAAAACACTG	57°C
(AJ786388)	84–115	3-HTBR2CD	115 bp	TCCCAGCCAGTGTTTTGACAAG	60°C
					
TβRIII, human	2501–2520	5-HTBR3E13	217 bp	TGTGTGCCTCCTGACGAAGC	59°C
(NM_003243)	2609–2717	3-HTBR3E15		AGGCTGCAAACGCAATGCCC	

TGF-β1, human	1402–1420	5-HTGFB1E3	426 bp	TGGCGATACCTCAGCAACC	55°C
(NM_000660)	1809–1827	3-HTGFB1E6		GTTGGCATGGTAGCCCTTG	
					
TGF-β2, human	680–699	5-HTB2CP	185 bp	CAACAGCACCAGGGACTTGC	65°C
(M19154)	845–864	3-HTB2CP		AGCACAAGCTGCCCACTGAG	
(NM_003238)	658–679	5-TGFB2E1B	272 bp	CCCCGGAGGTGATTTCCATCTA	62°C
	908–929	3-TGFB2E1B	188 bp	GTAGGGTCTGTAGAAAGTGGGC	
					
TGF-β3, human	342–361	5-TGFB3E1	332 bp	TGGACTTCGGCCACATCAAG	57°C
(NM_003239)	653–673	3-TGFB3E2		CTCCACTGAGGACACATTGAA	

GAPDH^d^, human	402-421	5-GAPDH	300 bp	CGTCTTCACCACCATGGAGA	59°C
(NM_002046)	682-701	3-GAPDH		CGGCCATCACGCCACAGTTT	

^*a*^Acc No, EMBL/DDBJ/GenBank Accession Number

^*b*^AT, annealing temperature

^*c*^this position is equivalent to 1–20 in AJ619019

^*d*^GAPDH, glyceraldehyde-3-phosphate dehydrogenase

### Alternative splicing and mRNA expression of TβRII

**Figure 2:**
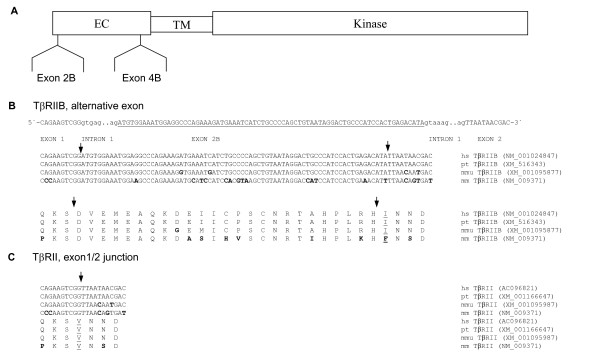
(**A**) Schematic drawing of the TβRII protein (EC, extracellular domain; TM, transmembrane domain; Kinase, Ser/Thr kinase domain) with the two alternatively spliced exons 2B and 4B. (**B**) Nucleotide sequence of the cDNA and deduced amino acid sequence of exon 2B (underlined capital letters) and splice site junctions (lower case letters) of the variant TβRIIB are shown. (**C**) Additionally, the partial nucleotide and amino acid sequence of TβRII without exon 2B is shown. Underlined amino acids indicate amino acid exchange at the splice site junction due to the alternative splicing. Bold letters mark the amino acid and nucleotide exchanges with respect to the human sequence. Arrows indicate the exon boundaries. (hs, homo sapiens; pt, pan troglodytes; mmu, macaca mulatta; mm, mus musculus).

An alternatively spliced exon between the first two exons of the TβRII gene was described for mouse and human [28-30] (Fig. 2A–C). The cassette type exon 2B consists of 75 bp and codes for 25 amino acids. Due to admission of exon 2B amino acid exchange occurs at the splice site junction between both receptor variants, from isoleucine to valine in human and from phenylalanine to valine in the mouse sequence (Fig. 2B,C). Exon 2B shows 14 nucleotide exchanges between the human and mouse sequence coding for 7 different amino acids. In contrast the nucleotide and amino acid sequence from Pan troglodytes is 100% identical to human, whereas the nucleotide sequence from Macaca mulatta demonstrated two different nucleotides, thus resulting in one amino acid exchange.

**Figure 3:**
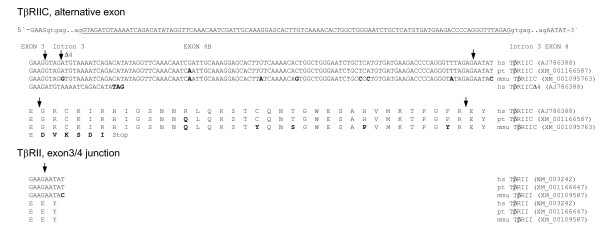
Nucleotide and amino acid sequence of exon 4B (underlined capital letters) of the variant TβRIIC and TβRIICΔ4 are given. Furthermore, the partial nucleotide and amino acid sequences of TβRII without exon 4B are shown. Bold letters mark the amino acid and nucleotide exchanges with respect to the human sequence. Arrows indicate the exon boundaries. (hs, homo sapiens; pt, pan troglodytes; mmu, macaca mulatta).

The alignment of the ESTs with the genomic sequence revealed the novel transcript TβRIIC (Acc. No. AJ786388) with the alternatively spliced exon 4B comprising 108 nucleotides arranged in frame and encoding 36 amino acids (Fig. 3). Exon 4B belongs to the cassette type of exons and is part of the extracellular domain of the receptor (Fig. 2A). The nucleotide and amino acid sequence from Pan troglodytes demonstrated one different nucleotide thus resulting in one different amino acid, whereas the nucleotide sequence from Macaca mulatta demonstrated 7 different nucleotides, thus resulting in 5 amino acid exchanges compared with the human sequence (Fig. 3).

**Figure 4:**
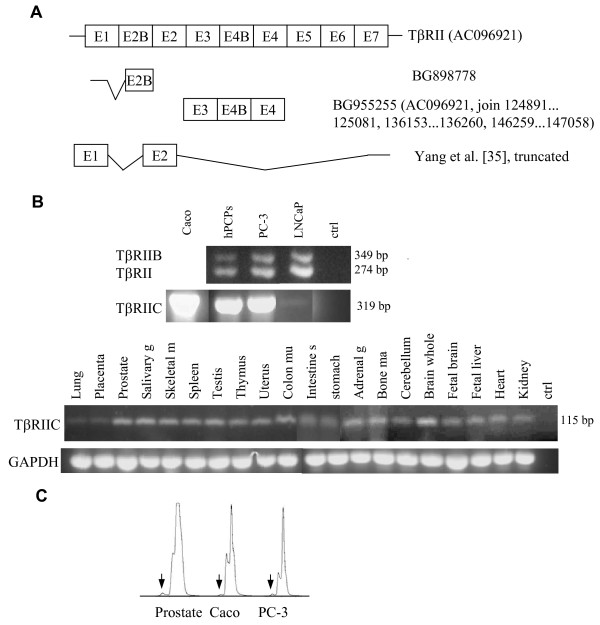
(**A**) Comparison of the exon structure of the human TβRII mRNA with the truncated sequence provided by Yang et al. [35]. Lines depict the 5'-UTR, 3'-UTR and ESTs with additional exons. (**B**) Expression pattern of both transcript variants of the TβRII gene in human prostatic cells (upper panel, 5-HTBR2B/3-HTBR2B). Expression of the novel splice variant TβRIIC in human prostatic cells (lower panel, nested PCR first round 5-HTBR2E3/3-HTBR2E4, second round 5-HTBR2Z/3-HTBR2E4) and normal human tissues (5-HTBR2E3/3-HTBR2CD) is shown. Additionally, GAPDH expression is also provided. (**C**) Fluorescence detection of TβRIICΔ4 (5-HTBR2E3/3-HTBR2CD, arrows) and TβRIIC is demonstrated. Caco, Caco-2; ctrl, control; g, gland; m, muscle; mu, mucosa; s, small; ma, marrow.

The alternatively spliced exon 2B was found in the human EST database (e.g. BG898778, Fig. 4A). We also analyzed the truncated TβRII sequence provided by Yang et al. [35] and found that it was identical to exon 1 and exon 2 and some nucleotides in the 3'-UTR, thus resulting in a truncated receptor isoform as published (Fig. 4A). Additionally, only one EST for TβRIIC could be identified (Fig. 4A). Expression of TβRII and TβRIIB was apparent in the prostate cells hPCPs, PC-3 and LNCaP (Fig. 4B). In contrast to the weak expression of TβRIB compared to TβRI, the long variant TβRIIB is as strongly expressed as TβRII. Because TβRIIC was very weakly expressed in the prostatic cell lines and the EST BG955255 was derived from colon tissue, we also analyzed the colon cancer cells Caco-2. With nested RT-PCR, mRNA expression could be detected in Caco-1, PC-3 and hPCPs, but only very weakly in LNCaP cells (Fig. 4B). Despite the low expression, we found mRNA expression in up to 20 normal tissues (Fig. 4B). Furthermore, we identified an aberrant splicing pattern at the 5'-end of the alternative exon of TβRIIC, where an alternative AG was used for splicing (Fig. 4C), resulting in a preliminary stop codon (Fig. 3). Although expression of TβRIICΔ4 was low compared to TβRIIC (Fig. 4C), it was apparent in all cell lines and tissues studied.

### Alternative splicing and mRNA expression of TβRIII (betaglycan)

**Figure 5:**
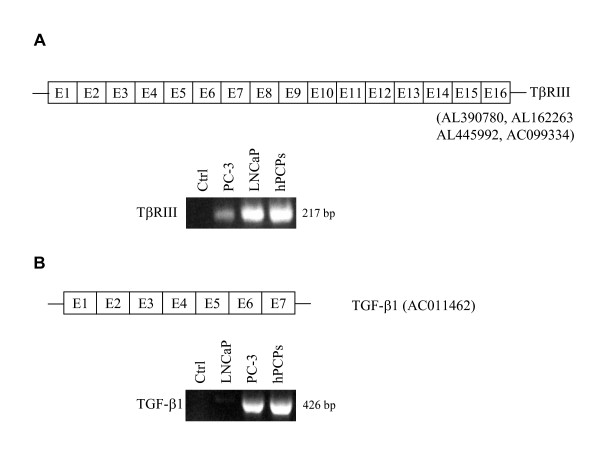
(**A**) Exon structure of the human TβRIII (betaglycan) mRNA. Lines depict the 5'-UTR and 3'-UTR. Expression pattern of the TβRIII gene in human prostatic cells (5-HTBR3E13/3-HTBR3E15). (**B**) Exon structure of the human TGF-β1 mRNA. Lines depict the 5'-UTR and 3'-UTR. Expression pattern of the TGF-β1 gene in human prostatic cells (5-HTGFB1E3/3-HTGFB1E6). Ctrl, control.

The alignment of the ESTs for TβRIII with the genomic sequence did not reveal additional exons. All prostatic cells expressed mRNA of the TβRIII (Fig. 5A).

### Alternative splicing and mRNA expression of TGF-β1

The alignment of the TGF-β1 gene with the ESTs did not show any new exons. Because in the TGF-β2 gene the alternatively spliced exon 2B between the first two exons was found as mentioned above, we tested whether this was also the case for the TGF-β1 cDNA. However, in the prostatic cells no additional exon was identified (data not shown). Besides LNCaP all cell lines studied showed expression of TGF-β1 (Fig. 5B). Additionally, we tested whether exons 4 and 5 were deleted in the human sequence as has been published for the porcine sequence [24]. However, in the prostatic cell lines studied this deletion was not detectable (Fig. 5B).

### Alternative splicing and mRNA expression of TGF-β2

**Figure 6:**
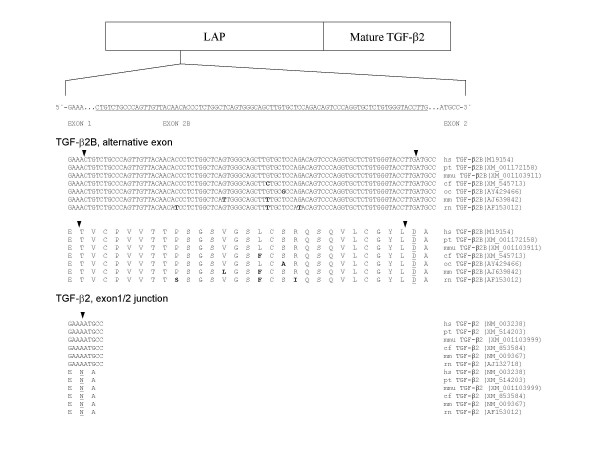
Schematic drawing of the TGF-β2 protein (LAP, latency-associated peptide) with the alternatively spliced exon 2B. Nucleotide and amino acid sequence of exon 2B (underlined capital letters) of the variant TGF-β2B are shown. Additionally, the partial sequence of TGF-β2 without exon 2B is shown. The sequence of TGF-β2 was not available for oryctolagus cuniculus. Underlined amino acids indicate amino acid exchange at the splice site junction due to the alternative splicing. Bold letters mark the amino acid and nucleotide exchanges with respect to the human sequence. Arrows indicate the exon boundaries. The accession numbers are also given. (hs, homo sapiens; pt, pan troglodytes; mmu, macaca mulatta; cf, canis familiaris; oc, oryctolagus cuniculus; mm, mus musculus; rn, rattus norvegicus).

For the TGF-β2 gene an additional cassette type exon between exons 1 and 2 was published for man and rat [12,25] and is now also available for monkeys, dogs, rabbits and mice (Fig. 6). The coding sequence is 84 bp in all species, resulting in additional 28 amino acids with a change from asparagine to aspartic acid in TGF-β2B at the splice site (Fig. 6). Nucleotide and amino acid sequences of TGF-β2B of the different species are more closely related to the human sequence than these of TβRIIC or TβRIIB. For example, the nucleotide and amino acid sequence of TGF-β2B from Pan troglodytes and Macaca mulatta is 100% identical to the human sequence (Fig. 6), whereas the nucleotide sequence of TβRIIC from Pan troglodytes and Macaca mulatta is only 99.1% and 93.5%, respectively, identical to the human sequence (Fig. 3).

**Figure 7:**
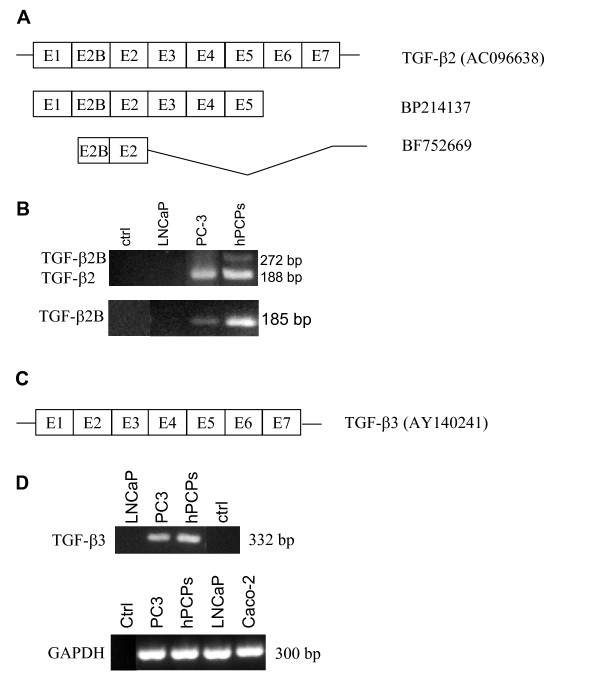
(**A**) Comparison of the exon structure of the human TGF-β2 mRNA with the ESTs BP214137 and BF752669 containing the additional alternative exon 2B. Lines depict the 5'-UTR, 3'-UTR and introns. (**B**) Expression of both transcript variants (upper panel, 5-TGFB2E1B/3-TGFB2E2B) and expression of the splice variant TGF-β2B (lower panel; 5-HTB2CP/3-HTB2CP) is shown. (**C**) Exon structure of the human TGF-β3 mRNA. Lines depict the 5'-UTR and 3'-UTR. (**D**) Expression pattern of the TGF-β3 gene in human prostatic cells (left panel, 5-TGFB3E1/3-TGFB3E2). Additionally, GAPDH expression of all cell lines studied is shown. Ctrl, control.

The alignment of the ESTs coding for TGF-β2 with the genomic sequence showed the EST BF725669 to contain an additional exon (Fig. 7A). The alternatively spliced TGF-β2B and TGF-β2 are expressed in PC-3 and hPCPs cells, but expression of TGF-β2B was weaker in comparison to TGF-β2 (Fig. 7B).

### Alternative splicing and mRNA expression of TGF-β3

The alignment of the TGF-β3 cDNA sequence with the EST database only yielded incorrectly spliced exons (Fig. 7C). We found an annotation for alternative splicing of TGF-β3 in the ASDB database [22]. Although the TGF-β3 gene could be found in this genomic clone, the alternative splicing does belong to the next gene, adjacent to TGF-β3. mRNA expression of TGF-β3 was investigated with primers located in exon 1 and exon 2 to test for possible new exons. However, we only observed one specific amplicon in all prostatic cell lines except for LNCaP cells (Fig. 7D).  Expression of the housekeeping gene glyceraldehyde-3-phosphate dehydrogenase (GAPDH) for all cell lines used is shown in Fig. 7D. 

### Localization of the alternative splice variants

**Figure 8:**
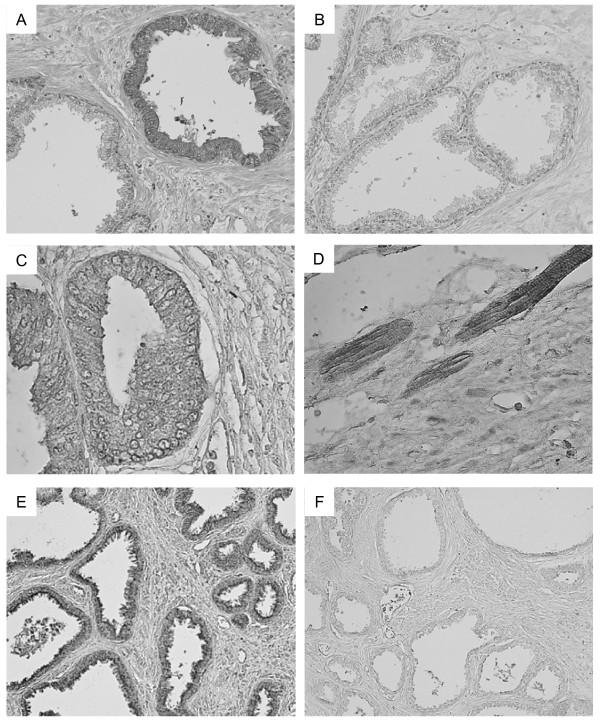
(**A**) Localization of TβRIIB in human prostate carcinoma is found in most epithelial cells (mainly in basal but also in columnar cells) in a nontumorous gland adjacent to a nontumorous gland without staining. (**B) **The negative control did not reveal any staining. Localization of TβRIIC in human prostate carcinoma was found in epithelial cells (**C**) and muscle cells (**D**). TGF-β2B was localized primarily in the apical region of epithelial cells (**E**). (**F**) The negative control did not reveal any staining. A-F, 100× magnification

Localization of the splice variant TβRIIB was found mainly in the basal cells but also in the columnar cells of the epithelium of nontumorous glands and is shown exemplarily for a prostate carcinoma patient with histological grading pT3apN0M0 (Fig. 8A). However, staining was also found in epithelial cells in tumorous glands (data not shown). The negative control without the primary antibody did not show any staining (Fig. 8B).

TβRIIC was localized in the epithelial cells (Fig. 8C) in very few glands of the human prostate (histological grading pT2apN0M0) and also in muscle cells (Fig. 8D, histological grading pT3bpN0M0). The splice variant TGF-β2B was found also mainly in the epithelial cells in the apical region (Fig. 8E, histological grading pT2bpN0M0). The negative control without the primary antibody did not show any staining (Fig. 8F).

### Functional analysis of the alternative splice variants

**Figure 9:**
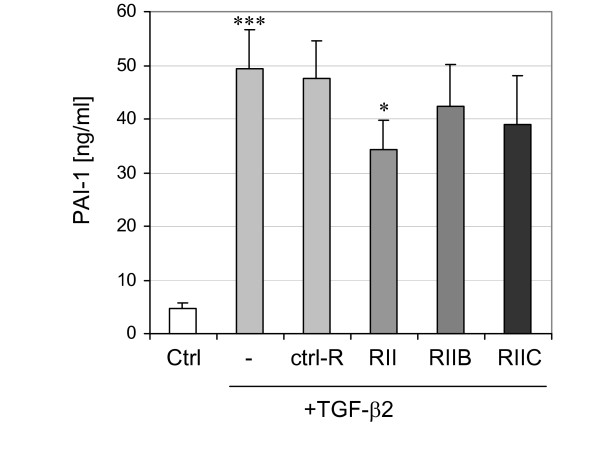
Secretion of PAI-1 by PC-3 cells was quantified by ELISAs. TGF-β2 alone (-) stimulated secretion of PAI-1 significantly compared to the control (Ctrl). Antibody perturbation experiments with antibodies specific for the extracellular domains of TβRII (RII), the alternative exons of TβRIIB (RIIB) and TβRIIC (RIIC), demonstrated a significant decrease in the amount of PAI-1 only for TβRII compared to the stimulation with TGF-β2 (-) or the unspecific antibody (ctrl-R). For the sake of clarity, we have not indicated that the antibody perturbation experiments were also significantly different to the control (Ctrl) without any TGF-β2 treatment. An unspecific antibody (ctrl-R) did not inhibit PAI-1 secretion stimulated by TGF-β2. Each experiment was independently repeated five times (n = 5) in duplicate, with each value given as the mean ± SEM. Statistically significant differences are indicated (*, *P *< 0.05; ***, *P *< 0.001).

We used a sensitive ELISA to determine the amounts of plasminogen activator inhibitor-1 (PAI-1) in the supernatant of PC-3 cells which are known to secrete PAI-1 [36]. Stimulation of PC-3 cells with 10 ng/ml recombinant TGF-β2 demonstrated a highly significant approximately 10-fold increase in PAI-1 secretion compared to the unstimulated control (Fig. 9). Antibody perturbation experiments performed with three different antibodies against TβRII, TβRIIB and TβRIIC showed a significant reduction (approximately 30%) in secretion of PAI-1 only for the anti-TβRII antibodies compared to the stimulation with TGF-β2 (Fig. 9). Although antibodies against TβRIIB and TβRIIC demonstrated a reduction of 14% and 21%, respectively, in PAI-1 secretion, the effects were not significant.

## Discussion

We have analyzed the mRNA expression of the TGF-betas and their respective high-affinity receptors in human prostatic cells available to us and identified alternative splicing in TβRI, TβRII and TGF-β2 (Table 2). It is striking that the alternatively spliced exons are located in the N-terminal part of the proteins, whereby in both receptors the additional amino acids were part of the extracellular domain. Also, the novel isoform TβRIIC is characterized by 36 additional amino acids in the extracellular domain. Up to date, the variant TGF-β2B is sequenced in more species and showed less sequence variations than TβRIIB and TβRIIC. Additionally, we demonstrated that the splice variants are translated and found a preferential epithelial localization in the human prostate. For the first time we could show that stimulation of PC-3 cells with TGF-β2 resulted in a significant and strong increase in secretion of PAI-1 with a major participation of TβRII and to a lesser extent that of TβRIIB and TβRIIC.

**Table 2:** Expression pattern of the TGF-β ligands, receptors and splice variants

	hPCPs	LNCaP	PC-3
**Ligands**			
TGF-β1	+	-	+
TGF-β2	+	-	+
TGF-β3	+	-	+

**Receptors**			
TβRI	+	+	+
TβRII	+	+	+
TβRIII	+	+	+

**Splice variants**			
TβRIB	+	+	-
TβRIIB	+	+	+
TβRIIC	+	-	+
TGF-β2B	+	-	+

### mRNA Expression of prostatic TGF-betas and their receptors

Analysis of mRNA expression of TGF-betas and their receptors in human prostatic cell lines showed very controversial results. It is generally accepted that PC-3 cells express TGF-β1, TGF-β2 and TGF-β3 [37,38] and even more the TGF-β2 gene was originally cloned from PC-3 cells [12]. In line with this, our study also showed expression of all TGF-beta ligands in the stromal hPCPs and PC-3. However, experiments with LNCaP yielded controversial results. Whereas mRNA expression for TGF-β1 to TGF-β3 was demonstrated [37] all other studies including this one could not find mRNA expression of TGF-β1 in LNCaP cells [39,40]. Our study showed expression of all TGF-beta ligands in the stromal cells hPCPs which is in accordance with the results for other stromal cell lines derived from the human prostate [41]. However, this study confirmed an earlier report showing that PC-3 cells express the splice variant TGF-β2B [12].

PC-3 cells showed expression of TβRI and TβRII, which was confirmed in this study. In LNCaP cells expression of TβRII was found, but TβRI was not expressed [42,43]. However, another study [44] like the present detected mRNA expression by RT-PCR in LNCaP cells. For stromal prostatic cells expression of TβRI and TβRII was found by us and others [45]. This is the first study to show expression of TβRIII and the receptor splice variants TβRIB and TβRIIB and to identify a novel transcript termed TβRIIC which will be discussed below. Only the stromal cells hPCPs expressed all receptor splice variants as well as TGF-β2B (Table 2). Except for the splice variant TβRIB, PC-3 cells also expressed all receptor splice variants, whereas LNCaP cells did not express TGF-β2B which is in line with the missing expression of all TGF-β ligands. Interestingly, these cells did also not express the newly identified splice variant TβRIIC (Table 2).

### Localization and protein data of the splice variants of TGF-β ligands and receptors

Agrotis et al. [30] have demonstrated, that TβRI is more abundant in contractile smooth muscle cells than the variant TβRIB. Additionally, they found that TβRI displayed a greater ability to induce PAI-1 mRNA in response to TGF-β1, whereas TβRIB performed slightly better in growth inhibition [30]. Interestingly, we identified for TβRI a pseudogene on chromosome 19, reaching from exon 2 to exon 4 and a short stretch of 62 bp from the 3'-UTR of the gene.

In contrast to TβRI which mainly is important for signal transduction, TβRII is involved in direct interaction with the ligands TGF-β1, TGF-β2 and TGF-β3 [1]. In TβRII the additional exon 2B was hypothesized to be involved in high-affinity binding of TGF-β2 to the receptor isoform TβRIIB also in the absence of TβRIII [10]. However, it was shown recently that TGF-β2 could bind to soluble TβRIIB or TβRII only in combination with soluble TβRI [46] and that also TGF-β1 could interact with TβRIIB [11]. Furthermore, the TβR knockouts, TβRI [47], TβRII [48] and TβRIII [49], revealed non-overlapping phenotypes with the TGF-β2 null mice [5], although TβRIII knockouts displayed reduced TGF-β2 binding [49]. This implies that either the high-affinity receptor for TGF-β2 is still not found or that receptor combinations might be responsible for the interaction.

Expression of TβRII was found in the human prostate in normal and tumor tissue primarily in the epithelial cells with a diminished expression in more advanced stages [16]. Similarily our results with TβRIIB also showed a distinct localization in the epithelial cells of normal and tumor tissue of the human prostate.

Our analysis clearly showed the expression of a novel transcript variant TβRIIC in PC-3, hPCPs cells, Caco-2 and up to 20 normal tissues including human prostate, indicating a ubiquitous expression in human organs. The additional and alternatively spliced exon encodes 36 amino acids located in the extracellular domain in close proximity to the transmembrane domain. Although the database search for protein domains revealed no similarities to other proteins or specific motifs, it is noteworthy, that the additional domain contains two additional cysteines which might be important for protein folding. Interestingly, we found a deletion of 4 bp at the 5'-end of the additional exon 4B in TβRIICΔ4, possibly resulting in a truncated receptor. Although expressed at a very low level, it was found in normal tissue and preliminary results suggest this to be also the case in tumor samples. Interestingly protein localization of TβRIIC was also found mainly in the epithelial cells of the human prostate but in very few glands.

The splice variant TGF-β2B mRNA was first described in the prostatic cell line PC-3 [12,50] and in rats in skeletal muscles, aorta, primary bronchus, heart, uterus, sciatic nerve, and spinal cord [25]. Additionally, TGF-β2B mRNA and protein were found in most somatic and germinal cells of mouse and rat [51]. TGF-β2B was also demonstrated to be secreted by BSC-40 cells from monkeys [52]. The additional exon of TGF-β2B is part of the LAP-domain which is important for correct secretion and inactivation of the mature C-terminal TGF-β2 dimer [8]. The alternatively spliced exon 2B contains 3 additional cysteine residues which might be important for the formation of cysteine bonds and therefore might influence protein folding. However, TGF-β2B is secreted and forms a latent complex with the LAP [52]. It is important to note that TGF-β2B is cleaved similarly to TGF-β2 and yields a mature monomer/dimer of exactly the same size as mature TGF-β2 [52]. Because only mature TGF-β2 binds to the receptor it is equal whether mature TGF-β2 is cleaved from the short TGF-β2 variant or long TGF-β2B variant. Whether the existence of the two different TGF-β2 LAP complexes is required for different binding to LTBPs and thus might be stored differently in the ECM warrants further investigation.

Up to date TGF-β2B was identified in most species, whereas TβRIB and TβRIIB were found in fewer species. It is noteworthy that TβRIIB is not as well conserved between human and mouse than TGF-β2B and up to date was not found in rat [51]. Therefore, we conclude that TβRIIB is not as ubiquitously expressed in the different species like the other variants and therefore could not serve as a ubiquitous receptor for TGF-β2. In line with this assumption, we could observe only a moderate decrease in PAI-1 secretion after inhibition of TβRIIB or TβRIIC after stimulation of PC-3 cells with TGF-β2. However, this is the first report showing a 10-fold increase of PAI-1 secretion in PC-3 cells after stimulation with TGF-β2.

## Conclusion

In general, mRNA expression of the TGF-β and TβR splice variants was more heterogeneous and weaker compared to the variants without the alternative exons. The variant TGF-β2B was identified in most species and is up to date the best conserved isoform among the various species. Similarly, the splice variant TβRIB was also found in many species in contrast to the isoforms TβRIIB and TβRIIC which showed a more restricted species distribution. This is the first report showing a distinct localization of TGF-β2B, TβRIIB and TβRIIC in the human prostate mainly in the epithelium.

## Methods

### Cell lines and tissues

The stromal cells hPCPs from the human prostate were propagated as described [33]. LNCaP and PC-3 cells were purchased from American Type Culture Collection (ATCC) and cultivated as published [53]. Colon cancer cell line Caco-2 was purchased from ATCC and kindly provided by Dr W.W. Franke (German Cancer Research Center, Heidelberg, Germany) and kept under standard conditions. Total RNA from 20 normal human tissues was purchased (Becton Dickinson, Heidelberg, Germany).

### RNA isolation, cDNA synthesis and RT-PCR

Total RNA from the cell lines was isolated with Trizol (Gibco BRL, Karlsruhe, Germany) according to the manufacturer's instructions. Total RNA of Caco-2 cells was isolated using RNAeasy isolation kit (Qiagen, Hilden, Germany) according to manufacturer's protocol. Reverse transcription was performed using 2 μg of total RNA and Omniscript (Qiagen), except for total RNA from Caco-2, which was reverse transcribed as described elsewhere [54]. Primers used for PCR are denoted in Table 1 and were intron-spanning to overcome genomic contamination. PCR was performed on a Hybaid Omnigene Thermocycler (MWG Biotech, Ebersberg, Germany) using mainly PanScript Taq polymerase (Pansystems, Aidenbach, Germany) as described [55]. Amplification with the primers 5-TGFB3E1/3-TGFB3E2 was performed with the Platinum Taq Polymerase (Invitrogen, Karlsruhe, Germany) according to the manufacturer's instructions. The first round of the nested PCR to clone TβRIIC was done with the primers 5-HTBR2E3/3-HTBR2E4 from which 20 μl were used for the second round with the primers 5-HTBR2Z/3-HTBR2E4. The other fragment of Tβ RIIC was also cloned after a nested PCR with the primers 5-HTBR2B/3-HTBR2CD in the first round and primers 5-HTBR2E3/3-HTBR2CD were used for the second round. The nested PCR was performed with the Qiagen Taq DNA Polymerase and solution Q (Qiagen) on a PTC100 cycler (Biozym, Germany). Amplification was carried out for 35 cycles, except for 5-GAPDH/3-GAPDH which was run for 25 cycles and 5-HTBR2E3/HTBR2CD which was run for 30 cycles. After an initial heating to 94°C for 4 min, each cycle consisted of denaturing at 94°C for 45 sec, annealing at the temperatures indicated in Table 1 for 45 sec and elongation at 72°C for 90 sec except for the last extension which lasted 5 min. PCR products were separated on agarose gels, extracted with Qiaex (Qiagen), subcloned into the pCR2.0 vector (Invitrogen) and subsequently sequenced by MWG Biotech and GENterprise (Mainz, Germany). Amplification with the Cy-5 labeled primer 5-HTBR2E3 with the primer 3-HTBR2CD to detect TβRIICΔ4 was performed as described [56], except that cDNA instead of genomic DNA was used. PCR fragments were separated on 8% polyacrylamide gels [56].

### Screening for alternatively spliced ESTs

The exon and intron pattern of the TGF-betas and their receptors was either found in the NCBI sequence database or determined by sequence comparison of the cDNAs with the genomic sequences by using the Blast tool. Each exon of the respective cDNAs was aligned with all available ESTs from human. Then, every EST was aligned with the genomic sequences to find alternatively/incorrectly spliced exons, which were analyzed for standard splice sites (GT-AG at the 5'- and 3'-end, respectively) and for a continuous open reading frame. Only good candidates which fulfilled both criteria were further analyzed by RT-PCR.

### Generation of polyclonal antibodies

Polyclonal antibodies directed against the peptide SFCSIQSQVLCGYLD of the alternative exon of the rat TGF-β2B (Fig. 6) and against the peptide IRHIGSNNRLQRSTC of the alternative exon of TβRIIC (Fig. 3) were raised in two rabbits respectively according to standard protocols (Coring, Gernsheim, Gemany) as published [51]. These peptide sequences are highly homologous in most species and did not show any homology to other proteins. Polyclonal antibodies were also affinity-purified on a sepharose column. Specificity of the antibodies was tested in ELISAs (CovAbtest, Coring) and western blots. Negative controls were performed with the preimmune serum and showed no binding.

### Analysis of localization of TβRIIB, TβRIIC and TGF-β2B

Polyclonal antibody against TβRIIB was purchased from R&D Systems (Wiesbaden, Germany) and diluted 1:50 for immunohistochemistry. Polyclonal antisera against TβRIIC and TGF-β2B were used at dilutions of 1:50 and 1:100. Negative controls were performed by omitting the primary antibodies. Immunohistochemistry was done with the Envision System from DAKO (Hamburg, Germany) according to the instructions of the manufacturer with DAB staining and HE counterstaining.

### PAI-1 ELISA and antibody perturbation

Quantitation of PAI-1 was performed with the highly sensitive PAI-1 Antigen ELISA Kit (Technoclone, Vienna, Austria), according to the manufacturer's instructions. PC-3 cells (50,000 cells/well) were seeded on 24-well plates and grown in DMEM (+10% FCS and antibiotics) at 37°C and 5% CO_2 _for 24 h. Then, medium was changed to DMEM containing the antibodies against TβRII (diluted 1:12.5, AF-241-NA, R&D Systems), TβRIIB (diluted 1:12.5, AF1300, R&D Systems), and TβRIIC (diluted 1:12.5). Control incubations were performed (i) without antibody, and (ii) by replacement of the antibodies by anti-goat IgG (1:12.5; Invitrogen, Karlsruhe, Germany). After incubation at 37°C and 5% CO_2 _for 1 h, TGF-β2 (10 ng/ml) was added. The cells were grown at 37°C for 72 h and collected supernatants were stored with protease-inhibitors (Complete Mini, Roche, Mannheim, Germany) at -20°C until the PAI-1 ELISA was performed.

### Statistics

All experiments were repeated independently at least three times in duplicate. Values from all experiments were used for calculation of the means and their respective standard errors of the mean (SEM). Statistical tests of one way analysis of variance (ANOVA) followed by the non-parametric test of Kruskal Wallis were used to determine significant differences between different experimental groups and the controls by using GraphPad Instat 3 (GraphPad, San Diego, USA). P values less than 0.05 were considered statistically significant.

## Abbreviations

Acc No, EMBL/DDBJ/GenBank Accession Number; ANOVA, analysis of variance; AT, annealing temperature; ATCC, American Type Culture Collection; bp, base pairs; Caco, Caco-2; cf, canis familiaris; Ctrl, control; ctrl-R (unspecific antibody); EC, extracellular domain; ECM, extracellular matrix; EST, expressed sequence tag; GAPDH, glyceraldehyde-3-phosphate dehydrogenase; g, gland; hs, homo sapiens; Kinase, Ser/Thr kinase domain; LAP, latency-associated peptide; LTBP, latent TGF-β binding protein; m, muscle; ma, marrow; mm, mus musculus; mmu, macaca mulatta; mu, mucosa; oc, oryctolagus cuniculus; PAI-1, plasminogen activator inhibitor-1; pt, pan troglodytes; rn, rattus norvegicus; s, small; SEM, standard error of the mean; ss, sus scrofa; TGF-β, transforming growth factor-beta; TβR, TGF-beta receptor; TM, transmembrane domain.

## Authors' contributions

LK was responsible for designing, analyzing, collating and interpreting data and drafting the manuscript. JAS performed the ELISAs and antibody perturbation experiments, MMK, LL and AS the cell culture and immunohistochemistry, EVB and HB the RT-PCR, and GH and RH contributed to the design of the study and to the manuscript. All authors have read and approved the final version.
